# Ultrastructure and localization of *Neorickettsia* in adult digenean trematodes provides novel insights into helminth-endobacteria interaction

**DOI:** 10.1186/s13071-017-2123-7

**Published:** 2017-04-13

**Authors:** Kerstin Fischer, Vasyl V. Tkach, Kurt C. Curtis, Peter U. Fischer

**Affiliations:** 1 Infectious Diseases Division, Department of Medicine, Washington University School of Medicine, St. Louis, MO 63110 USA; 2 Department of Biology, University of North Dakota, Grand Forks, ND 58202-9019 USA

**Keywords:** Digenan trematodes, *Neorickettsia*, Electron microscopy, Immunohistochemistry, Localization

## Abstract

**Background:**

*Neorickettsia* are a group of intracellular α proteobacteria transmitted by digeneans (Platyhelminthes, Trematoda). These endobacteria can also infect vertebrate hosts of the helminths and cause serious diseases in animals and humans. *Neorickettsia* have been isolated from infected animals and maintained in cell cultures, and their morphology in mammalian cells has been described. However, limited information is available on the morphology and localization of *Neorickettsia* in the trematode host.

**Methods:**

We used a *Neorickettsia-*infected strain of the model trematode *Plagiorchis elegans* to infect Syrian Golden hamsters to produce adult worms. Ultrastructure of *Neorickettsia* was assessed by transmission electron microscopy of high pressure freezing/freeze substitution fixed specimens. A *Neorickettsia* surface protein from *P. elegans* (*PeN*sp-3) was cloned and antibodies against the recombinant protein were used to localize *Neorickettsia* by immunohistochemistry.

**Results:**

Ultrastructural analysis revealed moderate numbers of pleomorphic endobacteria with a median size of 600 × 400 nm and characteristic double membranes in various tissue types. Endobacteria showed tubular membrane invaginations and secretion of polymorphic vesicles. Endobacteria were unevenly localized as single cells, or less frequently as small morula-like clusters in the ovary, Mehlis’ gland, vitelline follicles, uterus, intrauterine eggs, testis, cirrus-sac, tegument, intestine and the oral and ventral sucker. Examination of hamster small intestine infected with *P. elegans* showed many endobacteria at the host-parasite interface such as the oral and ventral sucker, the tegument and the excretory pore.

**Conclusions:**

We conclude that adult *P. elegans* trematodes carry *Neorickettsia* endobacteria in varying numbers in many tissue types that support vertical transmission, trematode to trematode transmission *via* seminal fluid, and possibly horizontal transmission from trematodes to vertebrate hosts. These means appear to be novel mechanisms of pathogen transmission by endoparasitic worms.

**Electronic supplementary material:**

The online version of this article (doi:10.1186/s13071-017-2123-7) contains supplementary material, which is available to authorized users.

## Background

A number of parasitic worm species of medical or veterinary relevance contain intracellular α proteobacteria of the order *Rickettsiales*. For example *Wolbachia* endosymbionts are crucial for development and reproduction of some filarial nematodes and are a target for novel anti-filarial chemotherapy in humans and domestic animals [1]. *Neorickettsia* are a different group of endosymbionts that infect some digenean trematode species. They are similar to *Wolbachia* in terms of genome reduction, number of protein-coding sequences and biochemical pathways. Both, *Wolbachia* and *Neorickettsia*, lack the ability for lipopolysaccharide (LPS) synthesis [2, 3]. However, *Neorickettsia* are not obligatory endosymbionts (like *Wolbachia*), they can infect mammalian cells, and may have mammalian vertebrate reservoirs. Furthermore, some *Neorickettsia* species have been shown to be highly pathogenic causing Sennetsu fever in humans (*N. sennestu*), Potomac horse fever (*N. risticii*) or salmon poisoning disease in dogs (*N. helminthoeca*) [4].

*Neorickettsia* have been detected in a considerable number of trematode species by PCR [5], but were rarely directly observed in their trematode hosts by microscopy. *Neorickettsia* have been repeatedly described morphologically using mammalian cell cultures and a typical ehrlichial growth cycle with elementary body, initial body and morula has been suggested [6–8]. Although some microorganisms have been observed in various tissues of digenean parasites before [9, 10] little is known about the morphology and cell cycle of *Neorickettsia* in its trematode host. Vertical transmission has been described and granular, bacteria-like structures have been detected in eggs of the bat trematode *Acantharium oregonense* using an antiserum of a horse infected with *N. risticii* [11]. In a recent study the same crude antiserum was used to label *Neorickettsia* in different life-cycle stages of the rodent flatworm *Plagiorchis elegans* [12]. Although all trematode life-cycle stages may carry *Neorickettsia*, the exact mechanisms for vertical transmission within trematode generations and horizontal transmission to the mammalian hosts are not clear.

Digenean trematodes of the genus *Plagiorchis* (Plagiorchiidae) are intestinal parasites of a variety of reptiles, birds and mammals and only accidentally infect humans [13]. *Plagiorchis elegans* is abundant in some areas in North America and Europe and they naturally carry *Neorickettsia. Plagiorchis elegan*s can be maintained in the laboratory in Syrian golden hamsters (*Mesocricetus auratus*) [5, 14, 15], therefore, this model system has been used to study *Neorickettsia* in trematodes. In the present study we have infected hamsters with *P. elegans*, and studied the ultrastructure and localization of *Neorickettsia* in adult trematodes. We cloned and expressed a *Neorickettsia s*urface protein and raised antibodies against the recombinant protein. These antibodies were used to localize *Neorickettsia* not only at the attachment sides of the trematodes, but also in the tissue surrounding the intestinal mucosa of the hamster.

## Methods

### Parasite collection

Numerous *Lymnaea stagnalis* snails infected with the digenean *P. elegans*, were collected in October of 2015 from a pond in Pennington County, Minnesota, USA. Cercariae shed by snails were screened for the presence of *Neorickettsia* endobacteria following a real-time PCR protocol targeting a 152-bp portion of the 3′ end of the heat shock protein coding gene, GroEL as described by Greiman et al. [15]. One of the snails proved to contain *P. elegans* with high level of *Neorickettsia* infection and it was selected for isolation of sporocysts as described previously [14]. In order to obtain adult digeneans, sporocysts of *P. elegans* containing numerous infective metacerariae were fed directly to two outbred Syrian golden hamsters (Charles River Laboratories, Wilmington MA, USA). After 20 days hamsters were humanely euthanized and their small intestines were examined for adult *P. elegans* (Additional file 1: Figure S1) under a stereomicroscope.

### Tissue fixation

Adult *P. elegans* (Additional file 1: Figure S1) were fixed for immunohistochemistry in 4% buffered formaldehyde for 1–5 days. Small pieces (1 cm) of hamster small intestine containing 3–5 attached *P. elegans* worms were fixed in Bouin’s solution (Sigma, St. Louis MO, USA) for 24 h and washed afterwards for 3–5 days using 70% ethanol. For electron microscopy, 10 worms were chemically fixed and 6 worms were physically fixed by high pressure freezing/freeze substitution (HPF/FS) as described below. Adult *P. elegans* were chemically fixed using 2% paraformaldehyde/ 2.5% glutaraldehyde (Polysciences Inc., Warrington PA, USA) as described previously [16]. Furthermore, for DNA or protein studies, *P. elegans* worms were directly snap-frozen in PBS at -80 °C.

For PCR detection of *Neorickettsia* in hamster tissue, pieces (~20 mg) of the small intestine (about 1 cm distance from an accumulation of worms), the spleen, the kidney and the heart were collected and snap-frozen. As negative control respective biopsies of an uninfected hamster were used.

### PCR detection of *Neorickettsia* in tissue samples

DNA was prepared form tissue samples using the DNeasy Blood and Tissue kit (Qiagen, Hilden, Germany) according to the instructions of the manufacturer. Each sample was tested in duplicate and 1 μl of template DNA was used. As positive controls either snails infected with trematodes (conventional PCR) or adult *P. elegans* (qPCR) containing *Neorickettsia* were used. For conventional PCR primers targeting *Neorickettsia* 16 rDNA (n16S-25 F and n16S-1500R) were used as described previously [5]. For quantitative real-time PCR primers and probes were designed targeting either a *Neorickettsia* suface protein or *P. elegans* 28S rDNA (Additional file 2: Table S1) and used under standard TaqMan conditions in QuantStudioFlex6 thermocycler (Applied Biosystems) system.

### Transmission electron microscopy

Following chemical fixation samples were washed, postfixed in osmium tetroxide (Polysciences Inc.), rinsed in dH_2_0, stained with uranyl acetate (Ted Pella Inc., Redding CA, USA), rinsed again in dH_2_0, dehydrated and embedded in Eponate 12 resin (Ted Pella Inc.). Sections of 95 nm were cut with a Leica Ultracut UCT ultramicrotome (Leica Microsystems Inc., Bannockburn IL, USA), stained with uranyl acetate/ lead citrate, and viewed on a JEOL 1200 EX transmission electron microscope (JEOL USA Inc., Peabody MA, USA) equipped with an AMT 8 megapixel digital camera (Advanced Microscopy Techniques, Woburn MA, USA). Figure plates were assembled using Microsoft Powerpoint 2010 and Adobe Photoshop Elements 8.0. Images were not computationally enhanced except for adjustments for brightness and contrast.

### High pressure freezing/freeze substitution fixation

Individual live flukes were placed in specimen planchettes containing 20% bovine serum albumin and processed as described previously [16]. Briefly, planchettes were high-pressure frozen in a Leica EM PACT2 high-pressure freezer (Leica Microsystems), transferred to freeze substitution medium (acetone containing 2% osmium tetroxide, 0.1% uranyl acetate, and 5% dH_2_0) under liquid nitrogen and placed in the Leica AFS, automatic freeze substitution system (Leica Microsystems). For freeze substitution, the samples were brought to -90 °C for 1 h, remaining at -90 °C for10 h, and subsequently warmed to -20 °C for18 h. Samples were placed at 4 °C for 30 min prior to washing with anhydrous acetone at room temperature, embedded in Eponate 12 resin, and sectioned and stained as described above.

### Cloning and expression of a *Neorickettsia* surface protein *N*sp*-3*

Because of our experience to detect *Wolbachia* endosymbionts in *Brugia malayi* worms by immunohistology using the *Wolbachia* surface protein 1 (w*Bm*0432, GenBank accession number AE017321) we used its amino acid sequence to search the genome of *N. risticii* (GenBank accession number CP001431) [2, 3]. We identified a sequence annotated as *N. risticii* surface protein 3 (*N*sp-3) as a member of the porin subfamily (GenBank accession number gi|340806186) and designed primers (*N*sp3-F: 5′-CAC CAT GAT AAA TAG AAA GTT CCT AGT GGG TG-3′ and *N*sp3-R: 5′-GAA TTA TGC ATT CAT AGG TAC CAT C-3′) to amplify an open reading frame of *N*sp-*3* from genomic DNA extracted from *Neorickettsia-*infected *P. elegans*. The PCR product was cloned into the pET Directional Topo Expression vector (Invitrogen, Carlsbad CA, USA) and the open reading frame of the plasmid insert was confirmed by DNA sequencing. BL21 cells were transformed with the plasmid and the protein was expressed according to the instructions of the manufacturer. The recombinant His-tagged *P. elegans N*sp-3 protein (r*PeN*sp-3) was purified using His-Select Cobalt Affinity Gel (Sigma) chromatography and used for further studies.

### Generation of mouse r*PeN*Sp-3 antisera

Two BALB/c mice were each immunized using 20 μg purified r*PeNs*p-3 protein in complete Freund’s adjuvant and boosted with 20 μg r*N*Sp3 in incomplete Freund’s adjuvant 14 days later. Immune sera were collected 21 days post initial immunization. Pre-immune sera of the same mice were collected before the initial immunization and used as negative controls.

### SDS PAGE and western blot

Total adult *P. elegans* protein was extracted and electrophoresed using 4–12% NuPAGE Bis-Tris minigels (Invitrogen) as described previously [17]. Western blot was performed with native, *Neorickettsia* infected *P. elegans* total worm protein extract or r*PeN*sp-3 antigen. Briefly, 10 μg of antigen per cm was separated on a 412% reducing gel, blotted onto a nitrocellulose membrane, blocked at room temperature (RT) for 30 min using 5% non-fat dry milk (BioRad, Hercules CA, USA) in PBS, and incubated with animal sera at a dilution of 1:100. Sera and secondary antibodies were diluted in PBS-tween and incubated at 37 °C for 1 and 2 h, respectively. After incubation with a secondary antibody (anti-mouse IgG [H + L], Promega, Sunnyvale CA, USA), blots were washed with PBS-tween, and antibody binding was detected using nitro-blue tetrazolium/5-bromo-4-chloro-3′-indolyphosphate substrate (Sigma).

### Immunohistology

The alkaline phosphatase anti-alkaline phosphatase (APAAP) technique was applied for immunostaining according to the recommendations of the manufacturer (Dako, Carpinteria CA, USA) and as described earlier [18]. Pre-immune mouse serum diluted 1:200 in PBS was used as negative control. Antisera of the mice immunized with r*PeN*sp-3 were used as primary antibodies. Sera were tested at dilutions (in PBS/0.1% triton-X) of 1:50, 1:100, 1:200, 1:250, 1:500 and 1:1,000. The dilution 1:250 was determined to deliver the best signal/background relationship and all further experiments were performed at that dilution. Rabbit-anti mouse IgG (1:25; Dako) was applied as secondary antibody and was bound to the APAAP complex. As substrate for alkaline phosphatase the chromogen Fast Red TR salt (Sigma) was used and hematoxylin (Merck, Darmstadt, Germany) served as counter-stain. Sections were examined using an Olympus-BX40 microscope (Olympus, Tokyo, Japan) and photographed with an Olympus DP70 microscope digital camera.

For fluorescent analysis Alexa Fluor 488 conjugated goat anti-mouse IgG (H + L) (1:200, Thermo Fisher Scientific, Carlsbad CA, USA) was used as a secondary antibody. Alexa Fluor 633 conjugated wheat germ agglutinin (WGA, Thermo Fisher Scientific) was used as membrane stain at 200 μg/ml for 10 min prior to mounting. For mounting Prolong Gold Antifade medium (Thermo Fisher Scientific) with DAPI was used. Wide field fluorescence microscopy (WFFM, Axioskop 2 MOT Plus, Zeiss Ontario CA, USA) with plan-apochromat 100× oil, 63× or 40× objectives was performed as described previously [18]. Wide-field fluorescence microscopy analysis was performed at the Washington University Molecular Microbiology Imaging Facility (http://micro.imaging.wustl.edu/).

## Results

### Ultrastructure of *Neorickettsia* in adult *P. elegans*

**Fig. 1:**
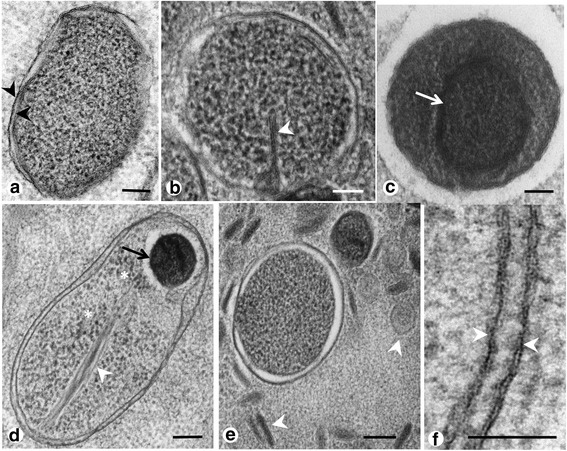
Single *Neorickettsia* endobacteria in adult, HPF/FS fixed *P. elegans*. **a** Coccoid endobacterium with characteristic double membranes (*arrowheads*). **b** Endobacterium with intracytoplasmatic membrane invagination (*arrowhead*). **c** Endobacterium with large halo, dark cytoplasm and electron-denser nucleoid (*arrow*). **d** Bacilloid endobacterium with membrane invagination (*arrowhead*), a small, dark nucleoid (*arrow*) and many free ribosomes (*asterisks*). **e** Endobacterium with tubular or vesicular structures (*arrowheads*) in close proximity. **f** Typical, electron-dense, double membrane (*arrowheads*) of *Neorickettsia* endobacteria. *Scale-bars*: 100 nm

**Fig. 2:**
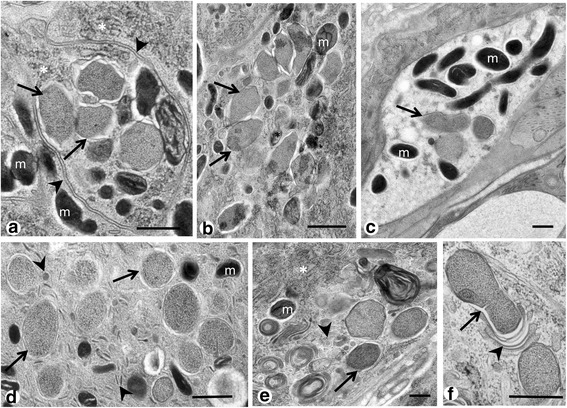
Cluster of *Neorickettsia* in *P. elegans* (HPF/FS). **a** Loose, morula-like cluster of endobacteria (*arrows*) surrounded by a double membrane (*arrowheads*) in proximity of the gut. Note the rough endoplasmatic reticulum in the trematode’s cytoplasm (*asterisks*). **b** Similar cluster of endobacteria (*arrows*) as in **a**, but without vacuole membranes. **c** Three endobacteria (*arrow*) in the vicinity of an accumulation of electron-dense mitochondria in the glycogen rich parenchyma. **d** Group of non-dividing endobacteria (*arrows*) in the tegument. Note the tubular or vesicular structures (*arrowheads*) between the endobacteria. **e** Endobacteria (*arrow*) in proximity of polymorphic vesicle structures (*arrowhead*) and rough endoplasmatic reticulum (*asterisk*). **f** Endobacterium (*arrow*) in binary fission. Note the different layers of membranes (*arrowhead*). *Abbreviation*: m, mitochondrion. *Scale-bars*: 500 nm

Endobacteria were highly pleomorphic in adult *P. elegans* (Fig. 1). Coccoid forms were usually between 300 and 500 μm in diameter, while bacilloid forms were up to 1500 μm long. Occasionally endobacteria showed a more electron dense nucleoid (Fig. 1c, d) and tubular invaginations of the double membrane (Fig. 1b, d). Tubular structures of up to 100 μm in length were sometimes observed within the cytoplasm of the endobacteria or in close proximity in the host’s cytoplasm (Fig. 1e). These elongated tubes were most likely produced and secreted by *Neorickettsia*. Endobacteria could be clearly differentiated from mitochondria in *P. elegans*, because the later were more electron dense, showed the characteristic cristae and contained insufficient double stranded DNA to be labeled by a specific antibody recognizing double stranded DNA (Additional file 3: Figure S2). Sometimes endobacteria were clustered in smaller morula-like structures (Fig. 2a, b), but overall single or small groups of 2–5 endobacteria were more frequently detected (Fig. 2c, e) and the typical reproduction was binary fission (Fig. 2f). A growth cycle with elementary bodies, initial bodies and large morulae was not observed in somatic worm tissues.

### Localization of *Neorickettsia* by TEM

**Fig. 3:**
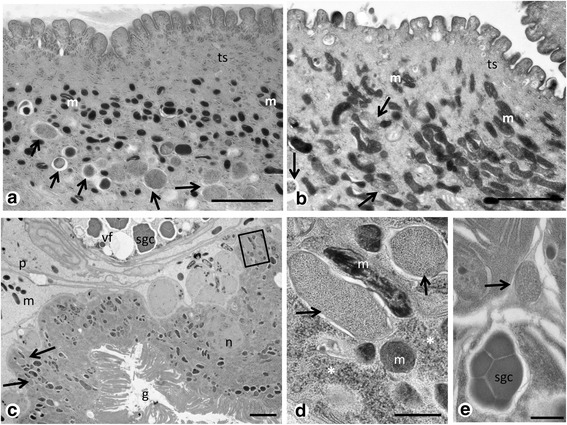
*Neorickettsia* in various tissues of adult *P. elegans*. **a** Endobacteria (*arrows*) in the tegument (HPF/FS). **b** Similar section as in **a**, but in a chemically fixed specimen. **c** Endobacteria (*arrows and square*) in the gut epithelium (HPF/FS). **d** Magnification of the squared area in **c** showing endobacteria (*arrows*) in proximity of mitochondria and ribosomes (*asterisks*). **e** Endobacterium (*arrow*) between vitelline cells within a follicle. *Abbreviations*: g, gut; m, mitochondrion; p, parenchyma; sgc, shell globule cluster; ts, tegumental syncytium; vf, vitelline follicle;. *Scale-bars*: **a**-**c**, 2 μm; **d**, **e**, 500 nm

**Fig. 4:**
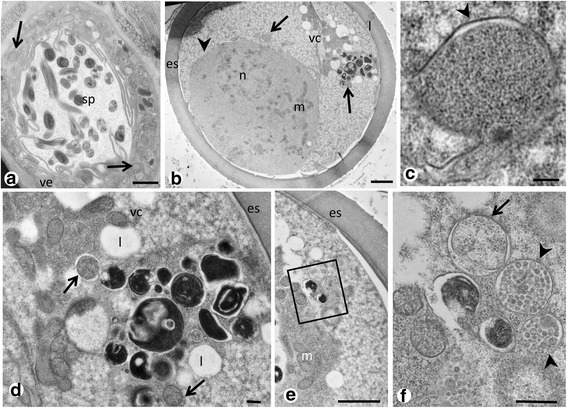
*Neorickettsia* in reproductive tissue of adult *P. elegans* (HPF/FS). **a** Cross-section through the vas efferens, note the endobacteria (*arrows*) in the inner tissue of the vas efferens. **b** Overview of a cross-section through an intrauterine egg showing the zygote (*arrowhead*) and the vitelline syncytium. Single endobacteria (*arrows*) are localized in the vitelline tissue. **c** Magnification from **b** showing the membranes (*arrowhead*) of an endobacterium. **d** Another magnification from **b** showing a few endobacteria (*arrows*) in close proximity of the vitelline material. **e** Small cluster of endobacteria in an intrauterine egg. **f** Magnification of the boxed area in **e** showing a large endobacteria in a vacuole (*arrow*) and two typical large *Ehrlichia*-like morulae (*arrowheads*) with many small reticulate cells. These typical morula stage *Neorickettsia* were observed only in developing eggs. *Abbreviations*: es, egg shell; l, lipid droplet; m, mitochondrion; n, nucleus; vc, vitelline cell. *Scale-bars*: **a**, **b**, **e**, 2 μm; **c**, **d**, 100 nm; **f**, 500 nm

Single or small clusters of endobacteria were commonly found in the proximal cytoplasm of the tegument (Fig. 3a, b), often in the vicinity of mitochondria and ribosomes (Fig. 3c, d). Density of endobacteria in the tegument varied by body region and no or few endobacteria were seen in the distal region of the tegument (Additional file 4: Figure S3a). Single or small clusters of endobacteria can be seen in many tissue types, including the reproductive tissue and tissue of the digestive system (compare Table 1, Fig. 3 and Additional file 4: Figure S3b, c). In the vitelline follicles single endobacteria were observed between vitelline cells (Fig. 3e). Small numbers of endobacteria were seen in the tissue of the vas efferens in close proximity to spermatozoa (Fig. 4a). For technical reasons intrauterine eggs of *P. elegans* with thick egg shells were not well preserved after HPF/FS or chemical fixation, but occasionally endobacteria were observed in the vitelline cells of intrauterine eggs (Fig. 4b-d). In one case we detected a few large morulae that contained many small reticulate cells in vitelline material of an intrauterine egg (Fig. 4e-f).

**Table 1:** Summary of the localization of *Neorickettsia* in adult *Plagiorchis elegans* trematodes as detected by transmission electron microscopy (HPF/FS fixation), immunohistochemistry (IHC) or immunofluorescence (IF)

Body parts	Endobacteria localization			Remarks
	HPF/FS	IHC	IF	
Body wall				
Peripheral tegument	+	++	++	Density-dependent on the location of the tegument
Inner tegument, subtegumentary perikaryon	+	+	++	
Parenchyma	+	+	+	
Digestive system				
Oral sucker	na	+	+	
Esophagus	na	+	+	
Ventral sucker	na	+	+	
Proximal caecum	++	+++	+++	
Distal caecum	++	+++	+++	
Reproductive system				
Seminal vesicle	na	na	++	
Ootype	na	++	++	
Ovary	na	+	+	
Vitelline glands	+	–	++	Mostly single bacteria
Uterus	–	–	–	
Eggs	+	+	++	Mostly single bacteria
Vas efferens	+	+	+	
Testis	–	++	++	
Spermatogonia	na	–	+	
Spermatocytes	na	–	+	
Mehlis’ gland	na	+++	+++	

*Key*: – not detected; + sometimes single endobacteria; ++ usually a few endobacteria; +++ many endobacteria, sometimes in high density

*na* not applicable/examined

Comparison of tissue sections fixed by glutaraldehyde or by HPF/FS revealed similar features, while membranes were usually better preserved by HPF/FS (Fig. 3a, b). Tubular membrane structures (as depicted in Fig. 1) were rarely observed in chemically fixed specimens (Additional file 5: Figure S4).

### Secretion of polymorphic vesicles

**Fig. 5:**
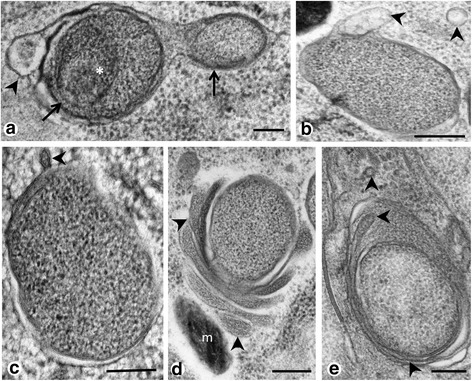
Secretion of polymorphic vesicles by *Neorickettsia* in adult *P. elegans*. **a** Two endobacteria (*arrows*) within one vacuole. Note the nucleoid (*asterisk*) and the membrane vesicle (*arrowhead*). **b** A single endobacterium secreting large membrane vesicles (*arrowheads*). **c** Endobacterium secreting a small, more electron-dense vesicle (*arrowhead*). **d** Endobacterium in close contact with stacks of elongated vesicles (*arrowheads*). **e** Endobacterium in proximity of elongated vesicles and stacks of membranes (*arrowheads*). *Abbreviation*: m, mitochondrion. *Scale-bars*: 200 nm

Like most gram-negative bacteria *Neorickettsia* secrete outer membrane vesicles. However, these vesicles are highly polymorphic reaching from small, 50–80 μm vesicles to large, 100–200 μm long round or oval structures, or elongated up to 300 μm long tubular structures (Fig. 5). In most examined worm tissue types *Neorickettsia* produced outer membrane vesicles, indicating extensive metabolic activity and interaction with the trematode host.

### Expression and characterization of *PeNsp*-3

**Fig. 6:**
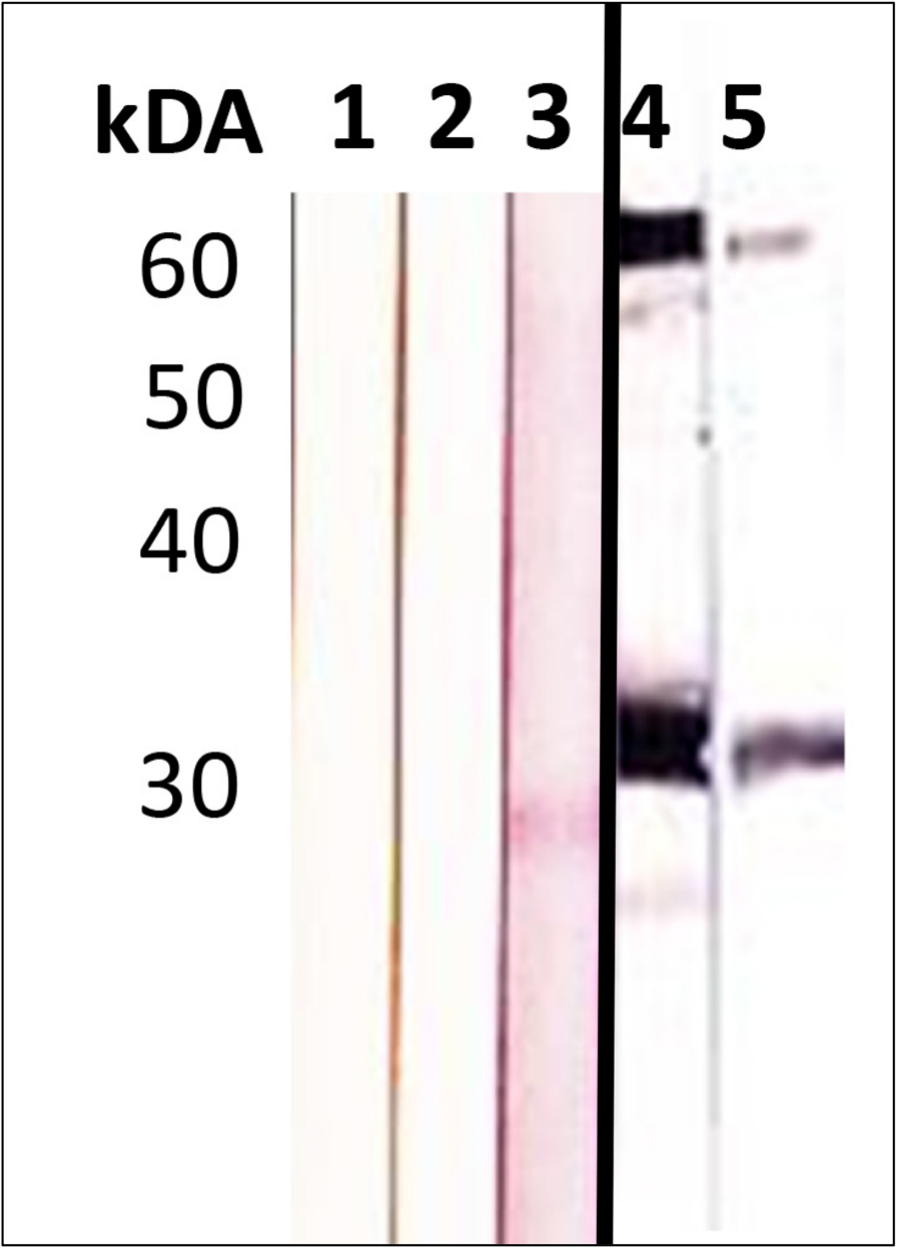
Western blot using native *P. elegans* total soluble worm extract (Lanes 1–3) or recombinant *P. elegans Neorickettsia* surface protein-3 (r*PeN*sp-3, Lanes 4, 5) as antigens probed with a mouse pre-immune serum (Lane 1), with a monoclonal antibody against a His-Tag (Lanes 2, 5), with a mouse serum immunized with r*PeN*sp-3 (Lanes 3, 4)

The *PeN*sp-3 sequence was amplified from genomic *P. elegans* DNA resulting in an open reading frame of 783 base pairs (GenBank accession number KX082665) with adapters for directional cloning in the expression vector. The recombinant, His-tagged, *PeN*sp-3 is comprised of 296 AA with a predicted molecular weight of 31.1 kDa, while the native protein has only 260 AA with a predicted MW of 27 kDA. These predictions were confirmed by western blot using either r*PeN*sp-3 or native total *P. elegans* extract as antigens and either a monoclonal antibody against the His-tag or a polyclonal mouse antibody against r*PeN*sp-3 as antibodies (Fig. 6). The His-tagged antibody recognized only the recombinant protein at the predicted MW, while the antibody against r*PeN*sp-3 recognized a relatively weak single band of ~27 kDA in the native worm extract and strong bands at ~31 and ~62 kDA in the purified recombinant protein. The larger band is most likely a dimer of the recombinant protein. The *PeN*sp-3 is 98% identical to *N*sp-3 from *N. risticii* Illinois [19]. The protein shows somewhat lower identity to *N*sp-3 of *N. sennetsu*, *Neorickettsia* from *Fasciola hepatica* and *N. helminthoeca*, while it shares only 30% identity to *W*sp-1, a surface protein of *Wolbachia* from the filarial nematode *B. malayi*.

### Immunohistochemical localization of *Neorickettsia*

**Fig. 7:**
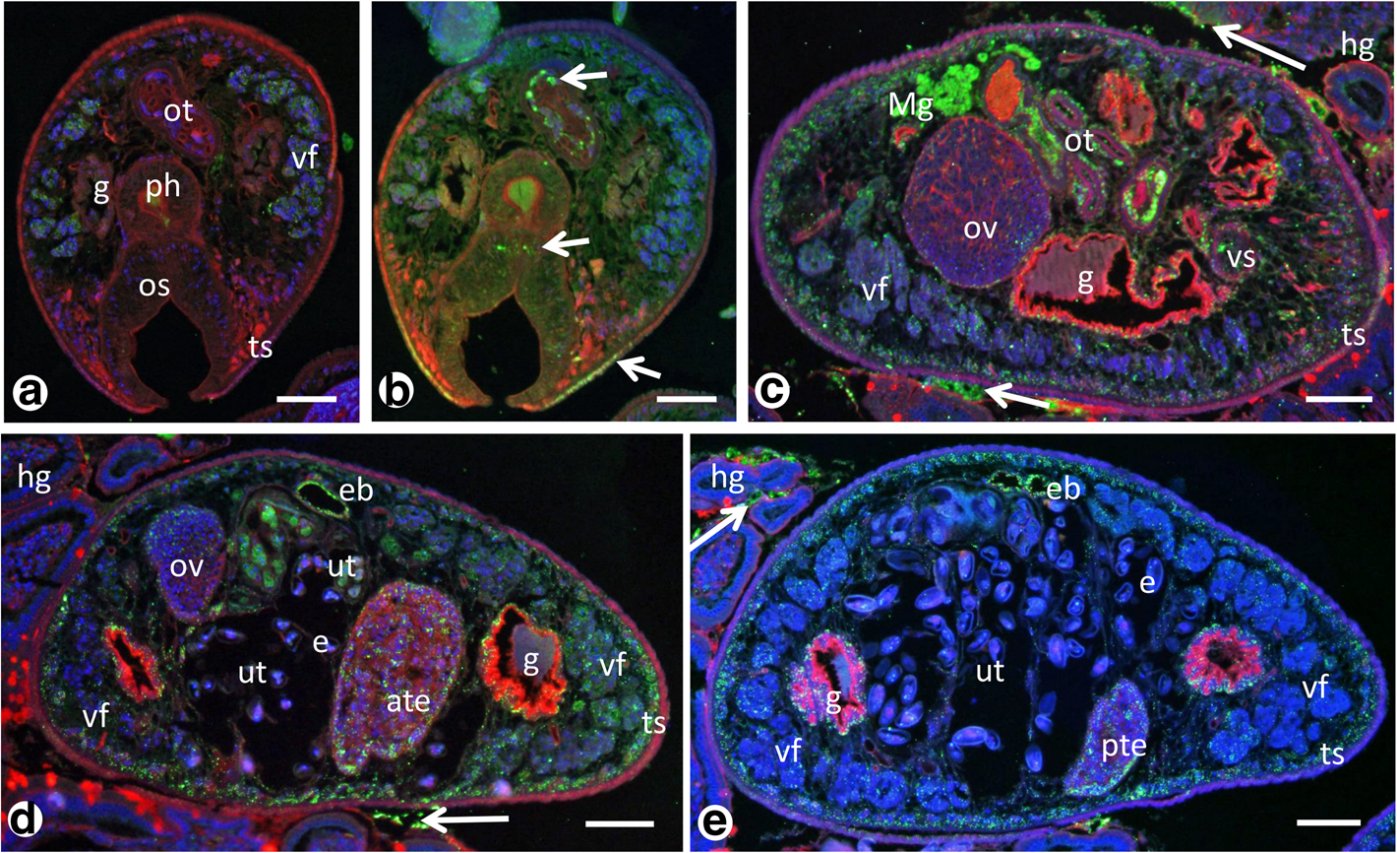
Overview of immunohistological detection of endobacteria in adult *P. elegans* using pre-immune serum (**a**) or polyclonal antisera against r*PeN*sp-3 (**b**-**e**). **a** Cross-section of the proximal part of the worm labeled with a pre-immune serum as negative control. Plasma membranes are stained in red and nuclei are stained in blue (DAPI). **b** Consecutive section to **a** stained for *PeN*sp-3. Note clusters of green endobacteria (*arrows*) in the tegument, the oral sucker and the ootype. **c** Cross-section of the region of the ventral sucker (compare Additional file 1: Figure S1) of an adult *P. elegans* in the intestine of a hamster stained for *PeN*sp-3. Numerous individual or small clusters of green-labeled endobacteria are seen in various worm tissues especially the tegument, the gut epithelium, ootype, vitteline glands and Mehlis’ gland. Note the green staining in the hamster gut (*arrows*). **d** Another cross-section of the mid-body region showing endobacteria also in the anterior testis and the epithelium of the excretory bladder. Again green labeling can be seen in hamster gut (*arrow*). **e** Cross-section of the distal part of an adult worm showing many endobacteria in the posterior testis, but only a few scattered endobacteria in the intrauterine eggs. Like in **c** and **d** green labeling of clustered endobacteria (*arrow*) can be detected occasionally in the hamster gut. *Abbreviations*: ate, anterior testis; eb, excretory bladder; e, egg; g, gut; hg, hamster gut; Mg, Mehlis’gland; os, oral sucker; ot, ootype; ov, ovary; ph, pharynx; pte, posterior testis; ts, tegumental syncytium; ut, uterus; vf, vitelline follicle; vs, ventral sucker;. *Scale-bars*: 100 μm

The antisera directed against r*PeN*sp-3 detected endobacteria with high sensitivity and little background (Fig. 7b-e), and the pre-immune sera used as negative control did not show any specific staining (Fig. 7a). Table 1 and Fig. 7 provide an overview of the endobacteria localization in *P. elegans*. In all examined specimens (*n* = 10), endobacteria were detected in somatic tissues as well as in female and male reproductive tissues. Although endobacteria appear to be randomly scattered throughout the worms, densities were highly variable in different tissue types but a clear distribution pattern was recognized.

### Localization of *Neorickettsia* in female reproductive organs and tissues

**Fig. 8:**
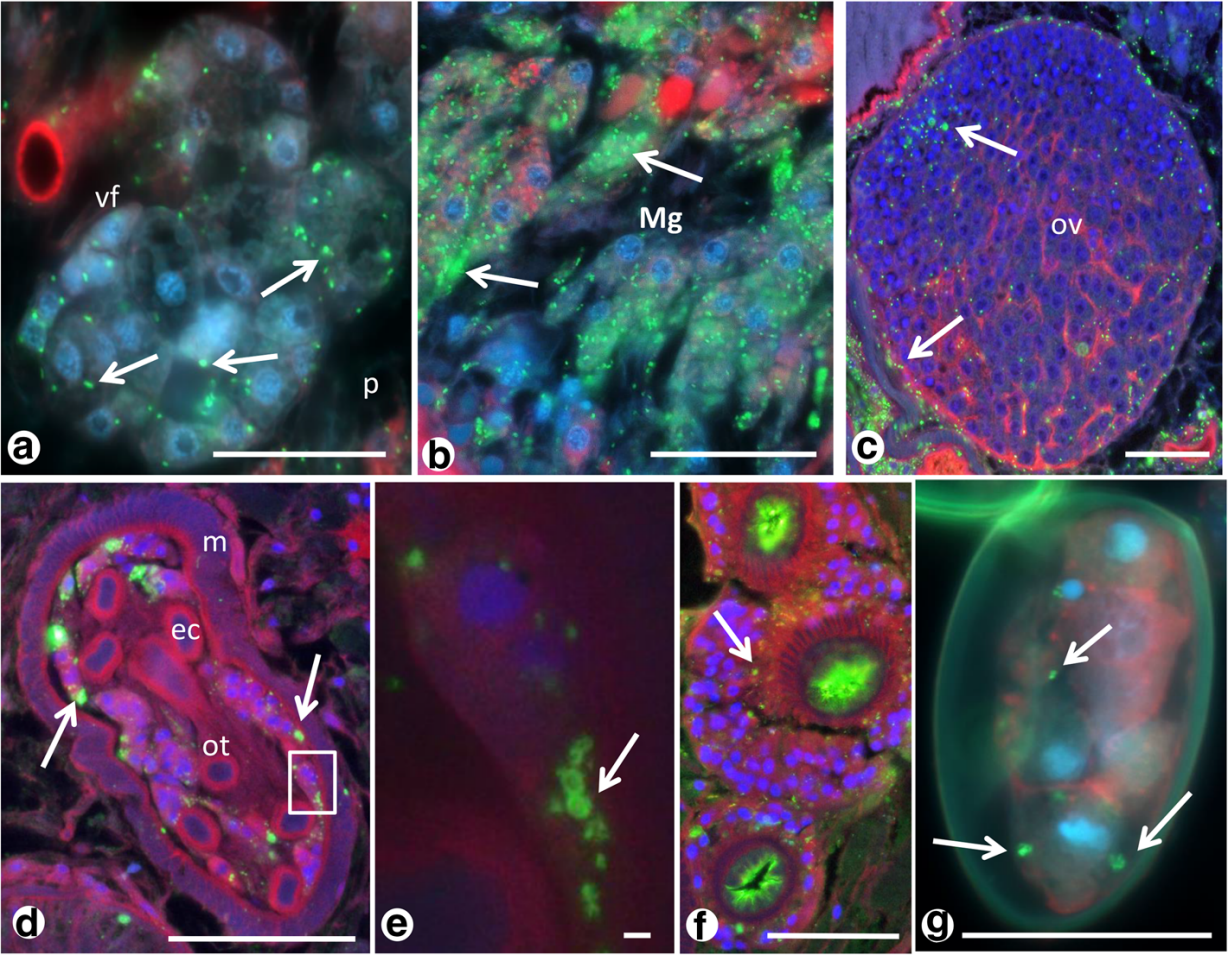
Immunohistological detection of endobacteria in female reproductive tissue of adult *P.elegans* using antisera against r*PeN*sp-3. **a** Single endobacteria (*arrows*) in the vitelline follicles. **b** High density of endobacteria (*arrows*) in the Mehlis’ gland. **c** Endobacteria (*arrows*) in the ovary, with low numbers in the center and moderate density in the periphery. **d** Clusters of endobacteria (*arrows*) in vitelline material within the ootype. No endobacteria were labeled in the egg cells. **e** Magnification of boxed area in **e** showing the typical ‘donut’- shaped staining pattern (*arrow*) for a surface protein. **f** Endobacteria (*arrow*) in the metraterm. The cross-section of the distal uterus shows green autofluorescence. **g** A few endobacteria (*arrows*) in a fully assembled intra-uterine egg. Note that the egg shell shows weak green autofluorescence. *Abbreviations*: ec, egg cell; m, membrane; Mg, Mehlis’ gland; ot, ootype; ov, ovary; p, parenchyma; vf, vitelline follicle. *Scale-bars*: **a**, **c**-**e**, 25 μm; **f**, **g**, 1 μm

Moderate numbers of endobacteria per host cell were observed in the vitelline follicles (Fig. 8a), but given the abundance of vitelline follicles the total number of *Neorickettsia* in vitelline tissue is high. High densities of endobacteria were observed in the Mehlis’ gland (Fig. 8b), but this gland is relatively small compared to other tissues. Some endobacteria were also seen in the ovary, but mostly in its periphery (Fig. 8c). In the ootype, where the yolk-rich trematode egg is ‘assembled’, endobacteria are rarely seen, but rather in the vitelline follicles (Figs. 3e and 8d). Endobacteria were also observed in the metraterm, the distal portion of the uterus close to the genital atrium (Fig. 8f). In the intrauterine eggs only a few endobacteria were detected (Fig. 8g), and sometimes no endobacteria were seen.

### Localization of *Neorickettsia* in male reproductive tissue

**Fig. 9:**
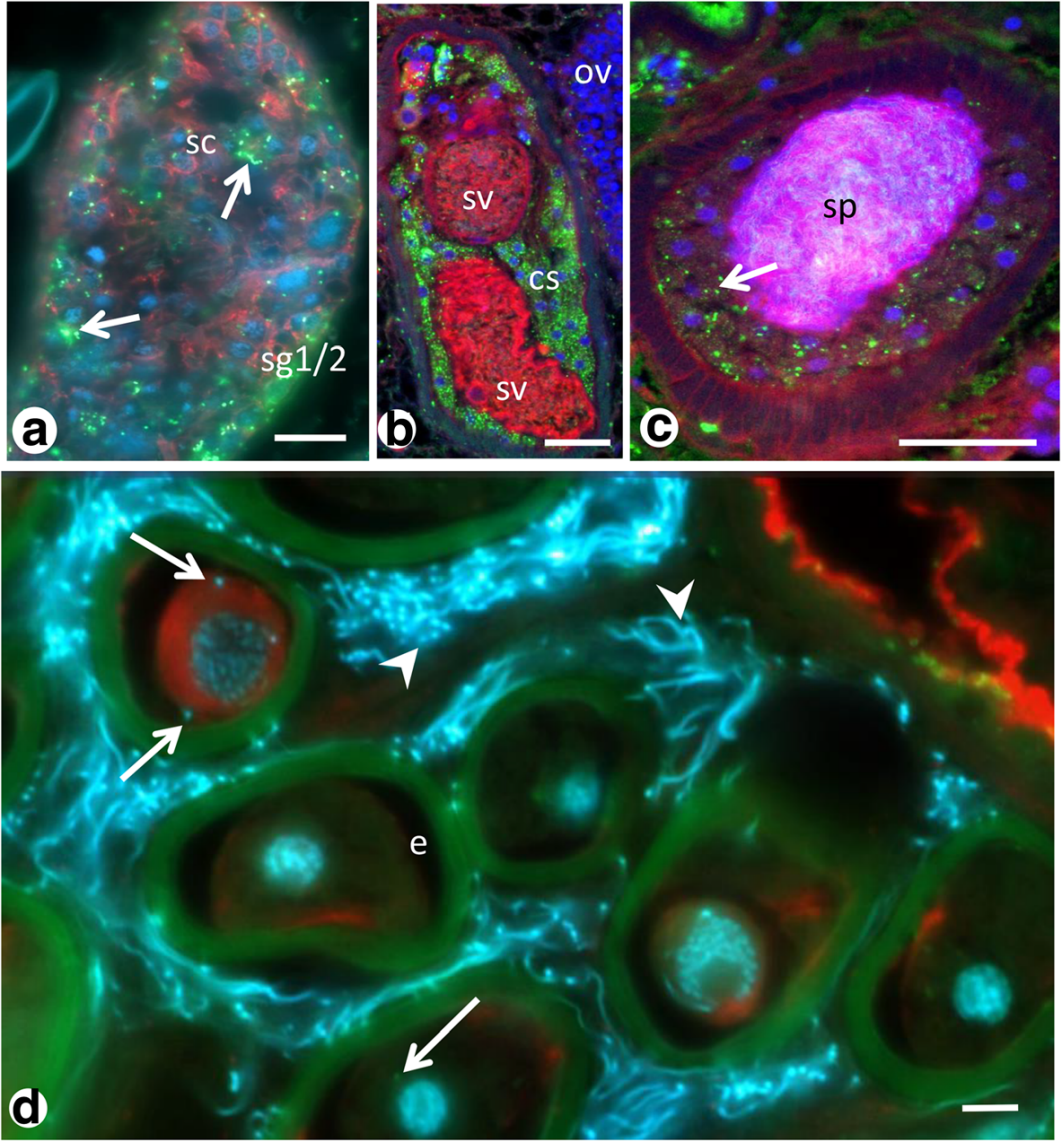
Immunohistological detection of endobacteria in male reproductive tissue of adult *P. elegans*. **a** Endobacteria (*arrows*) in testis. **b** Many endobacteria in the cirrus sac. **c** Endobacteria (*arrow*) in tissue within the muscular cirrus sac. Note the large amount of spermatozoa in the center. **d** Endobacteria (*arrows*) in the cytoplasm of an intrauterine egg. Note the numerous spermatozoa (*arrowheads*) migrating towards the ootype and the weak green autofluorescence of the eggshell. *Abbreviations*: cs, cirrus-sac; e, egg; sc, spermatocytes; sg1/2, primary and secondary spermatogonia; sp, spermatozoa; sv, seminal vesicle. *Scale-bars*: 1 μm

Endobacteria were surprisingly abundant within the male reproductive tissues. In the testis endobacteria were seen in the spermatogonia and the spermatocytes (Fig. 9a). Furthermore, endobacteria were detected in tissue of the vas efferens, the seminal vesicle and the cirrus-sac (Fig. 9b, c). No endobacteria were seen in intrauterine spermatozoa that were inoculated together with seminal fluid during mating, but single endobacteria were detected in the cytoplasm of eggs in the same region (Fig. 9d).

### Comparison of *Neorickettsia* detection using APAAP and immunofluorescence

**Fig. 10:**
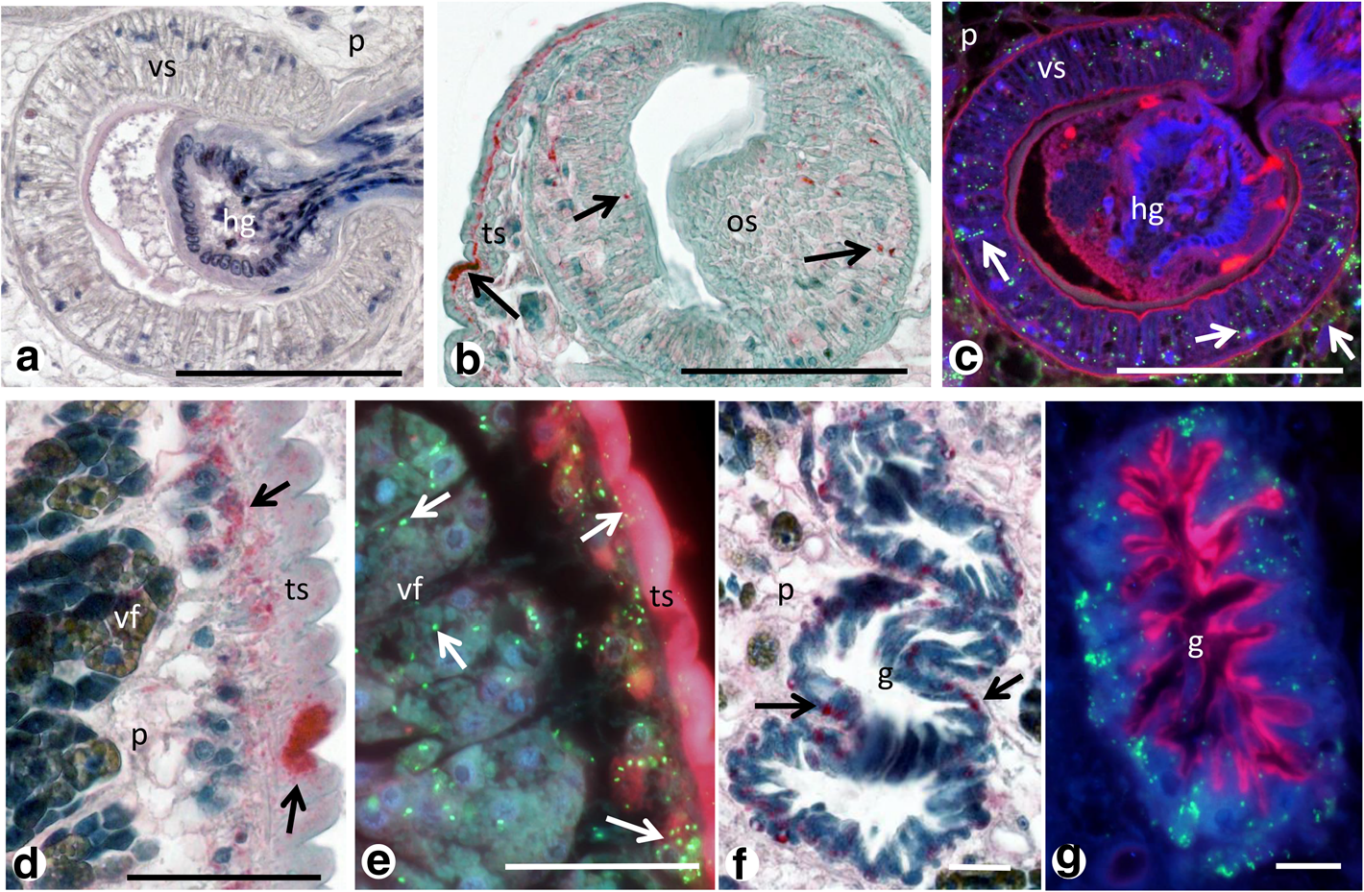
Comparative immunohistological detection of endobacteria using the APAAP method (**a**, **b**, **d**, **f**) and immunofluorescence (**c**, **e**, **g**). **a** Section of the ventral sucker stained with a pre-immune serum showing no red labeling for endobacteria. **b** Another section of a sucker showing red labeled endobacteria (*arrows*) in the sucker and the surrounding tegumental syncytium and parenchyma. **c** Consecutive section to **a** showing distinct labeling of endobacteria (*arrows*) in the sucker and parenchyma with high resolution by immunofluorescence. **d**, **e** Endobacteria (*arrows*) in the tegument and vitelline follicles. Note the more sensitive detection in **e. f**, **g** Endobacteria (*arrows*) in the outer epithelium of the trematode gut. *Abbreviations*: g, gut; os, oral sucker; p, parenchyma; ts, tegumental syncytium; vf, vitelline follicle; vs, ventral sucker. *Scale-bars*: **a-c**, 100 μm; **d**, **e**, 25 μm; **f**, **g**, 10 μm

We used two different immunohistology techniques to localize *Neorickettsia* in adult *P. elegans* using a polyclonal mouse antibody against r*PeN*sp-3. While the APAAP method detects not only the endobacteria, standard counterstain provides also information on the general histological morphology of *P. elegans* (Fig. 10a, b, d, f, Additional file 6: Figure S5). In contrast, immunofluorescence microscopy provides a higher resolution, but additional fluorescence dyes or antibodies are needed to accurately localize the endobacteria relative to worm organs or tissues (Fig. 10c, e, g). For example due to higher resolution and sensitivity, a higher number of endobacteria was detected in the tegument by immunofluorescence compared to the immunohistochemistry using APAAP (Table 1).

### Detection of *Neorickettsia* in hamster tissue

The presence of endobacteria in the suckers and the intestinal tract (Figs. 7b-e and 10b, c, f, g) could indicate a direct transmission during attachment to the host tissue and/or feeding. Another possibility of transmission is the excretion of *Neorickettsia* through the excretion pore, since large numbers of endobacteria were detected in the excretory bladder (Fig. 7d, e) or *via* shedding of the tegument, because *Neorickettsia* were frequently detected in the tegument (Fig. 10d, e). We have frequently detected *Neorickettsia* outside of *P. elegans* in the gut of a trematode-infected hamster (Fig. 7c-e, Additional file 7: Figure S6b-f). Therefore we screened different hamster tissues (heart, kidney, spleen, small intestine) for the presence of *Neorickettsia* DNA by conventional PCR, and we detected at this stage of infection *Neorickettsia* only in the intestine (Additional file 7: Figure S6g). The examined samples of the intestine did not contain any adult *P. elegans*. Although we did not detect any *P. elegans* eggs in the intestine by microscopy, it is possible that eggs were the source of *Neorickettsia* DNA. Therefore we screened the tissue samples and adult *P. elegans* for *Neorickettsia* DNA by qPCR. While, *Neorickettsia* DNA was only detected in the intestine and adult *P. elegans* (Additional file 8: Table S2) we did also detect *P. elegans* DNA in the intestine. However the relation of *Neorickettsia* DNA to trematode DNA was much higher in the gut compared to isolated *P. elegans*. For isolated worms almost 5 more amplification cycles (32-fold difference) were needed to detect *Neorickettsia* DNA compared to worm DNA. For the intestine of infected hamsters almost the same number of cycles (28.93 *vs* 28.18) was needed to detect *Neorickettsia* and worm DNA. These results can be interpreted as further evidence that the bacteria are not only residing in a few *P. elegans* eggs but are also infecting the intestine.

## Discussion

The present study is the first detection of *Neoricketttsia* in adult trematodes on the ultrastructural level. *Neorickettsia* have been shown to infect a broad variety of trematode parasites of wildlife [5]. Only recently *Neorickettsia* endobacteria have been shown to infect the common liver fluke *Fasciola hepatica* [20]. This important food-borne trematode infects about 2.6 million people and causes an estimated 3.2 billion US$ in annual losses in livestock animals. Therefore, a better understanding between trematode parasites and *Neorickettsia* endobacteria, that may constitute important pathogens themselves, is urgently needed.

High pressure freeze/freeze substitution fixation was well suited for TEM detection of *Neorickettsia* in *P. elegans*. Almost all previous studies on the ultrastructure of *Rickettsiales* used exclusively chemical fixation, and only few studies used physical fixation for TEM [16, 21]. While membranes and the electron-dense nucleoid were usually better preserved compared to chemical fixation the overall results were similar. The ultrastructural morphology of *Neorickettsia* in adult *P. elegans* described in the present study was in general similar to the ultrastructure of *N. risticii* and *N. sennetsu* described from mammalian cell cultures [22]. For example the formation of polymorphic tubules by the cytoplasmic membrane or the cell wall membrane has been described previously by Popov et al. [7]. However, the arrangement of *Neorickettsia* in *P. elegans* appears to be more similar to *N. sennetsu* that rarely forms large morulae with many reticulate or dense-cored cells like *N. risticii* [7, 8]. In our study large morulae were only occasionally observed in intrauterine eggs of *P. elegans*. Although related tick-transmitted *Ehrlichia* species may show different morphology in mammalian and tick cell cultures, the number of bacterial cells per morula is usually similar in vertebrate and vector cells [23, 24]. Therefore, our results indicate that propagation of *Neorickettsia* in adult *P. elegans* is predominantly of the *N. sennestu*-type.

We have successfully expressed *PeN*sp-3 and showed that polyclonal mouse antisera raised against *PeN*sp-3 are suitable for sensitive and specific detection of *Neorickettsia* in *P. elegans*. With this antibody endobacteria show the typical ‘donut’-shaped labeling pattern of a surface protein (Fig. 8e), while intensity of labeling may vary. *PeN*sp-3 is a distant ortholog of *W*sp-1 of *Wolbachia* and of the P28 outer membrane protein (Omp) of *Ehrlichia chaffeensis* [18, 25]. In tick and mammalian cell cultures of *E. chaffeensis* it has been shown that P28 Omp expression is tightly regulated with peaks of expression in the mid- and late-exponential growth phases at 28 and 37 °C [25]. Therefore it can be hypothesized that *Neorickettsia* may not show maximum expression of *PeN*sp-3 in all life-cycle stages of *P. elegans*. However, adult trematodes live at mammalian body temperature where *PeNsp*-3 may show its maximum expression level. Recently, a serum of a horse infected with *N. risticii* has been used to localize *Neorickettsia* in different life-cycle stages of *P. elegans* [12]. While our observations with the *PeN*sp-3 antisera confirm most of the results for adult trematodes from this previous study, minor differences were recorded that may be explained by the higher specificity and lower background staining observed in our staining using antisera against a single specific surface protein. For example, we unambiguously detected small numbers of endobacteria in periphery of the ovary.

Immunohistochemistry was used to localize *Neorickettsia* in entire histological worm sections in a larger number of *P. elegans* specimens. Transmission electron microscopy and IHC provided similar results (see Table 1), but fewer specimens were examined by TEM and endobacteria were not always detected the same tissues in both methods. We have especially analyzed the tissue distribution of *Neorickettsia* in many individuals with regard to its consequences for vertical and horizontal transmission. *Neorickettsia risticii* has been described to be vertically transmitted in its trematode host by the detection of endobacteria in eggs [11]. We have shown that unlike *Wolbachia* in filarial nematodes [18] the number of endobacteria in the mature ovaries of *P. elegans* is low and most endobacteria find their way into the egg in the ootype during ‘assembly’ of the yolk-rich egg. We also consider the possibility of transmission of *Neorickettsia* from trematode to trematode *via* mating, since male reproductive tissue is often heavily colonized. Pathogen transmission by spermatophores or seminal fluid during mating (venereal transmission) has been described previously from several other invertebrates [26, 27]. Adult *P. elegans* may not only act as vectors for *Neorickettsia*, but also function as reservoir with circulation of endobacteria between adult trematodes. On the other hand, some *Neorickettsia* species infect more than one digenean host species. Our findings do not explain how *Neorickettsia* are transmitted between digeneans belonging to different species. This is likely to involve horizontal transmission via tissues of either intermediate or definitive hosts.

It has been reported that *N. risticii* was horizontally transmitted to bats by its trematode host *Acantharium oregonense* or that *N. helminthoeca* was transmitted to dogs by adult *Nanophyetus salmincola* trematodes [11, 28]. In both reports trematode eggs were considered as source of infection. To study *N. helminthoeca* transmission by trematode eggs, dogs were injected either with fluke eggs homogenized in a glass tissue grinder or with intact eggs. Only homogenized eggs were infectious to dogs, suggesting that infectious *Neorickettsiae* were localized within the interior of the unembryonated *N. salmincola* eggs but not outside the egg shells [28]. In agreement with this observation we never observed in the present study endobacteria on the surface of the egg shell, and only relatively small numbers within the egg. Therefore, the mechanism of horizontal transmission is still unclear.

Adult *P. elegans* reside in the small intestine, produce eggs with thick egg shells like many other trematodes, and the eggs need the operculum to be opened to release miracidia. It appears unlikely that a considerable number of the sturdy and acid-resistant eggs burst and release *Neorickettsia* that will infect the mammalian host. Therefore, we do not favor the hypothesis that *Neorickettsia* are transmitted horizontally by trematode eggs. Tick-transmitted *Rickettsiales* are usually localized in large densities within the salivary glands of the vectors and transmitted with the saliva during the blood meal [29]. Little is known about the ‘saliva’ in trematodes, but given the fact that adult *P. elegans* are actively attached to the host tissue by their suckers (frequently causing a hemorrhage at the attachment site) and that *Neorickettsia* can be found in the suckers and the intestinal epithelium it is possible that they are actively transmitted at the attachment site. Other potential routes of horizontal transmission are during shedding of tegumental material or *via* the excretion pore. This first study indicates that 20 days after exposure of hamsters to *Neorickettsia*-positive *P. elegans* metacercariae, *Neorickettsia* infection outside the trematode is mainly restricted to the small intestine, the mucus and the connective tissue within the villi. Further studies have to elucidate the detailed mechanism of horizontal transmission.

It is generally agreed that *Neorickettsia* are not obligatory endosymbionts of digeneans, since neither all strains nor all individuals within a host species are usually infected [4, 15, 30]. The adult digeneans used in the present study were produced by a single snail infected with *Neorickettsia-*positive sporocysts and the examined adult worms were all infected with *Neorickettsia*. Our study showed that endobacteria are probably interacting with trematode tissues and organelles by releasing vesicles. However, it is not known whether *Neorickettsia* cause certain phenotypes such as *Wolbachia* endobacteria in insects. Since *Neorickettsia* are susceptible to some common antibiotics further studies may show whether removal of endobacteria by antibiotic treatment causes a phenotype in previously infected digeneans [31].

Taken together our study uncovers for the first time the ultrastructure of *Neorickettsia* in its trematode host, it establishes a novel detection method for *Neorickettsia* using *PeN*sp-3 antisera and describes in detail the localization of *Neorickettsia* in adult trematodes. The gathered information is crucial for proper explanation of the transmission mechanisms of *Neorickettsia* and to further analyze the relationship of *Neorickettsia* and their trematode host.

## Conclusions

Transmission electron microscopy of *Neorickettsia* in adult *P. elegans* reveals a high degree of pleomorphy. Polyclonal anti-sera raised against recombinant *PeN*sp-3 protein are helpful for the localization of *Neorickettsia*. These endobacteria are localized in varying numbers in many tissue types that support vertical transmission, trematode to trematode transmission via seminal fluid, and possibly horizontal transmission from trematodes to vertebrate hosts. These means appear to be novel mechanisms of pathogen transmission by endoparasitic worms.

## Additional files

Additional file 1: Figure S1. Overview of adult *P. elega*ns anatomy, showing the proportions of major organs and tissues. **a** Representative longitudinal section. **b** Whole, unstained, living trematode. **c** Magnification of the proximal part (boxed area from **b**) showing many anatomical details. *Abbreviations*: ate, anterior testis; cs, cirrus-sac; g, gut; me, metraterm; Mg, Mehlis’ gland; os, oral sucker; ov, ovary; ph, pharynx; pr, prostate gland; pte, posterior testis; sv, seminal vesicle; ut, uterus; vf, vitelline follicles; vs, ventral sucker. *Scale-bars*: 1 mm. (TIF 5591 kb)

Additional file 2: Table S1. Primers and probes used to detect *Neorickettsia* DNA and *P.elegans* DNA in various tissue samples by qPCR. (DOCX 15 kb)

Additional file 3: Figure S2. Immunogold labeling using an anti- DNA antibody which detects double stranded DNA in the nucleus and endobacteria, but not in mitochondria of *P. elegans*. **a** Intense labeling of electron dense areas of the nucleus of a subtegumental cell. **b** DNA of endobacteria (arrows) is extensively labeled by gold particles, while mitochondria that contain only about 1% of the amount of DNA are not labeled. **c** Three strongly labeled endobacteria (arrows) in a vacuole (arrow heads). **d** Small, more electron-dense endobacterium labeled for DNA in the cytoplasm (arrow) and close proximity inside and outside of the vacuole membrane (arrowhead). *Abbreviations*: m, mitochondrion; n, nucleus. *Scale-bars*: **a-c**, 500 nm; **d**, 100 nm. (TIF 6472 kb)

Additional file 4: Figure S3. TEM of HPF/FS fixed *P. elegans*. **a** Overview of a cross-section of the tegument showing a lose cluster of endobacteria (arrows) in one area, but no endobacteria in other areas. **b** Large endobacteria (arrows) are localized in the wall of the gut, while endobacteria are mostly absent in adjacent tissues. Note the cluster of small structures (boxed area) in the parenchyma. **c** Magnification of boxed area from **b** shows a few small endobacteria (arrows) with typical membrane structures among electron dense structures without pronounced membranes. *Abbreviations*: m, mitochondrion; sgc, shell globule cluster. *Scale-bars*: **a**, **b**, 5 μm; **c** 200 nm. (TIF 6120 kb)

Additional file 5: Figure S4. TEM of chemically fixed *P. elegans*. **a** Large clusters of endobacteria in the tegument (circles) and the cytons. **b** Large clusters of endobacteria (circles) in the subtegument. **c** Magnification of **b** showing pleomorphic endobacteria without well preserved membranes. **d** Three endobacteria with multiple and well preserved membranes. **e** Magnification of boxed area of **d** showing the well preserved membrane structure. *Abbreviation*: tsp, tegumental spine. *Scale-bars*: **a**, **b**, 2 μm; **c-e**, 500 nm. (TIF 6604 kb)

Additional file 6: Figure S5. APAAP staining for endobacteria in *P. elegans* using polyclonal antisera against r*PeN*sp-3. **a** Longitudinal section near the ventral sucker showing diffuse red staining for endobacteria in many tissue types. **b** Light pink background around the vitelline follicles (compare to Fig. 8a). **c** Similar to **b** ovary appears to be endobacteria free (no red stain), notice the red staining outside of the ovary (arrow). **d** Intense red staining for endobacteria in the Mehlis’ gland where density of *Neorickettsia* is high (compare to Fig. 8a). **e** Intense, but limited red staining for endobacteria in the testis (compare to Fig. 9a). **f** Intense red staining for endobacteria in the seminal vesicle and cirrus sac (compare to Fig. 9b). *Abbreviations*: ate, anterior testis; cs, cirrus-sac; g, gut; me, metraterm; Mg, Mehlis’ gland; ov, ovary; p, parenchym; sv, seminal vesicle; ut, uterus; vf, vitelline follicle; vs, ventral sucker. *Scale-bars*: **a**, **c**, **e**, **f** 100 μm; **b**, **d**, 25 μm. (TIF 7473 kb)

Additional file 7: Figure S6. Detection of *Neorickettsia* outside *P. elegans* (**a-d** APAAP staining, **e-f** immunofluorescence). **a** Adult *P. elegans* in the intestine of a hamster, pre-immune serum. **b** Consecutive section to **a** but stained with anti-sera against *PeN*sp-3. **c** Magnification of **b** showing connective tissue of the gut within the pharynx of *P. elegans.* Note single red cells (arrow) positive for *PeN*sp-3. **d** Another magnification of **b** showing single red cells (arrow) positive for *PeN*sp-3 in connective tissue within the hamster villi. **e** Green labeling for *PeN*sp-3 of bacteria (arrow) within the intestinal mucus or connective tissue within the villi. **f** High resolution, donut-shaped labeling for *PeN*sp-3 of bacteria (arrow) in the mucus. **g** Conventional PCR detection of *Neorickettsia* targeting 16S rDNA. Lane M: Marker; Lane 9: no template; Lanes 10, 11: *Neorickettsia* positive trematodes in field-collected snails; Lanes 1–4: *P. elegans*-infected hamster 1; Lanes 5–8: *P. elegans*-infected hamster 2; Lanes 12–15: uninfected hamster. Lanes 1, 5, 12: heart tissue; Lanes 2, 6, 13: kidney tissue; Lanes 3, 7, 14: spleen tissue; Lanes 4, 8, 15: small intestine tissue. *Abbreviations*: ct, connective tissue; os, oral sucker; ph, pharynx; ts, tegumental syncytium; vil, villi of the intestine. *Scale-bars*: 100 μm. (TIF 7014 kb)

Additional file 8: Table S2. Comparison of qPCR amplification (mean cycle threshold values, CT) of *P.elegans* DNA and *Neorickettsia* DNA from 20 mg of various tissues. Both targets were only detected in isolated *P. elegans* worms and in the small intestine from hamsters infected with *P. elegans* (but without adult trematodes present). Significantly more *Neorickettsia* DNA relative to trematode DNA was found in the intestine. (DOCX 14 kb)

## Abbreviations

HPF/FS: High pressure freezing/ freeze substitution
IF: Immunofluorescence
IHC: Immunohistochemistry
*PeN*sp-3: *Neorickettsia* surface protein-3 from *P. elegans*
