# Supplementary figures and images for: Ultrastructure and localization of Neorickettsia in adult digenean trematodes provides novel insights into helminth-endobacteria interaction
Source: Parasit Vectors. 2017 Apr 13;10:177. doi: 10.1186/s13071-017-2123-7 (PMC5390476; doi:10.1186/s13071-017-2123-7)

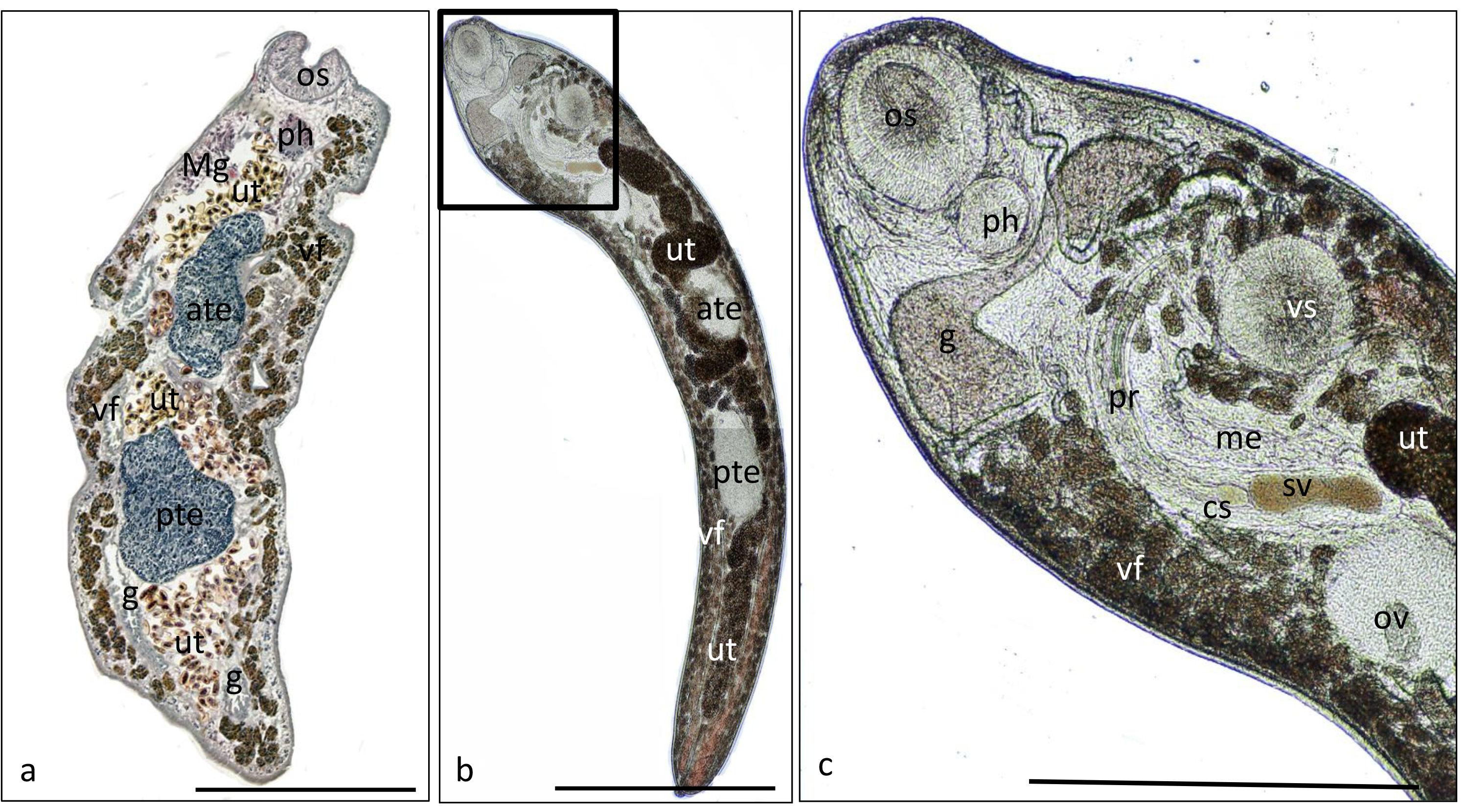

Supplement: Supplementary file 1 — Overview of adult P. elegans anatomy, showing the proportions of major organs and tissues. a Representative longitudinal section. b Whole, unstained, living trematode. c Magnification of the proximal part (boxed area from b) showing many anatomical details. Abbreviations: ate, anterior testis; cs, cirrus-sac; g, gut; me, metraterm; Mg, Mehlis’ gland; os, oral sucker; ov, ovary; ph, pharynx; pr, prostate gland; pte, posterior testis; sv, seminal vesicle; ut, uterus; vf, vitelline follicles; vs, ventral sucker. Scale-bars: 1 mm. (TIF 5591 kb) [file 13071_2017_2123_MOESM1_ESM.tif]

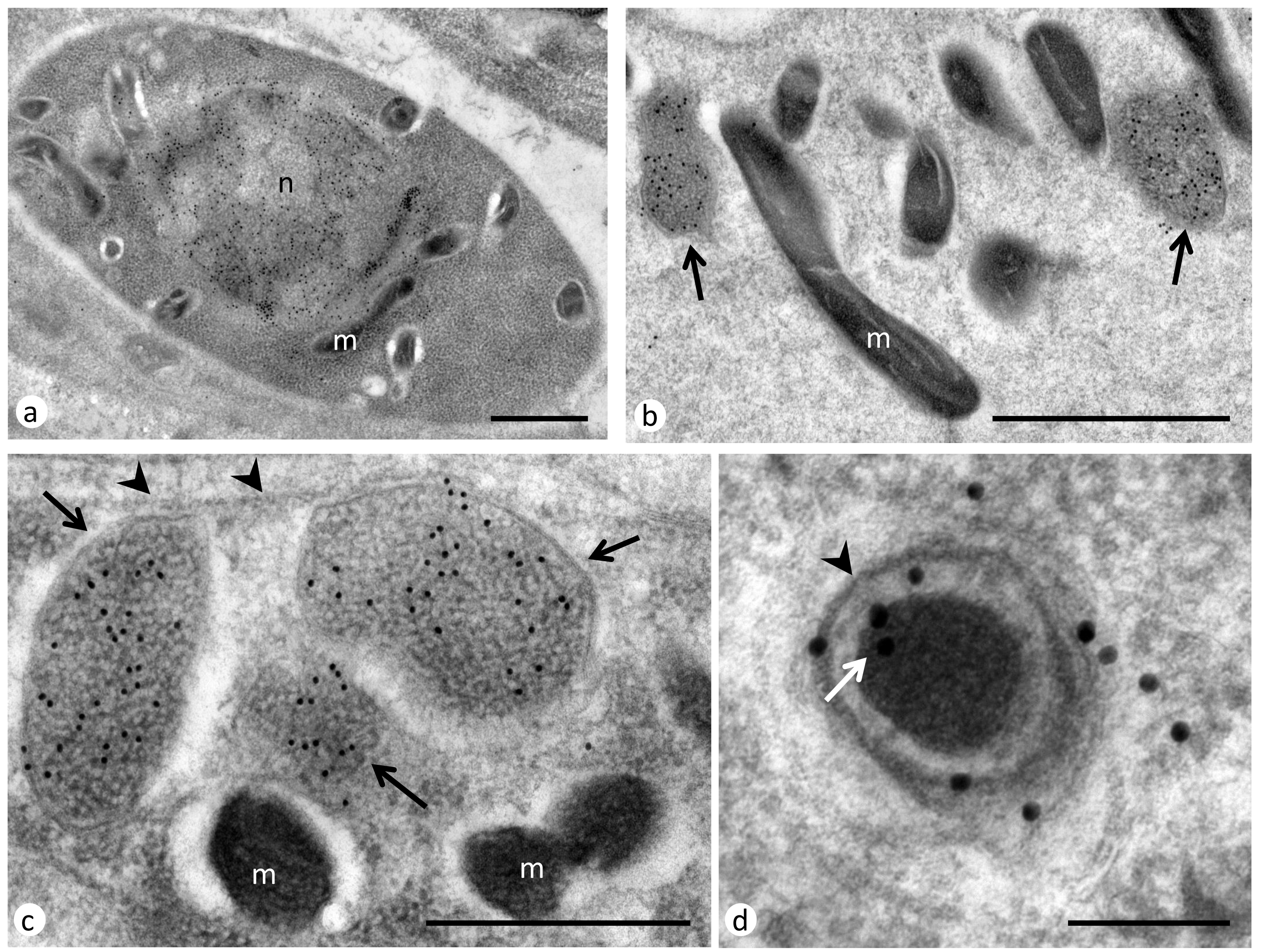

Supplement: Supplementary file 3 — Immunogold labeling using an anti- DNA antibody which detects double stranded DNA in the nucleus and endobacteria, but not in mitochondria of P. elegans. a Intense labeling of electron dense areas of the nucleus of a subtegumental cell. b DNA of endobacteria (arrows) is extensively labeled by gold particles, while mitochondria that contain only about 1% of the amount of DNA are not labeled. c Three strongly labeled endobacteria (arrows) in a vacuole (arrow heads). d Small, more electron-dense endobacterium labeled for DNA in the cytoplasm (arrow) and close proximity inside and outside of the vacuole membrane (arrowhead). Abbreviations: m, mitochondrion; n, nucleus. Scale-bars: a-c, 500 nm; d, 100 nm. (TIF 6472 kb) [file 13071_2017_2123_MOESM3_ESM.tif]

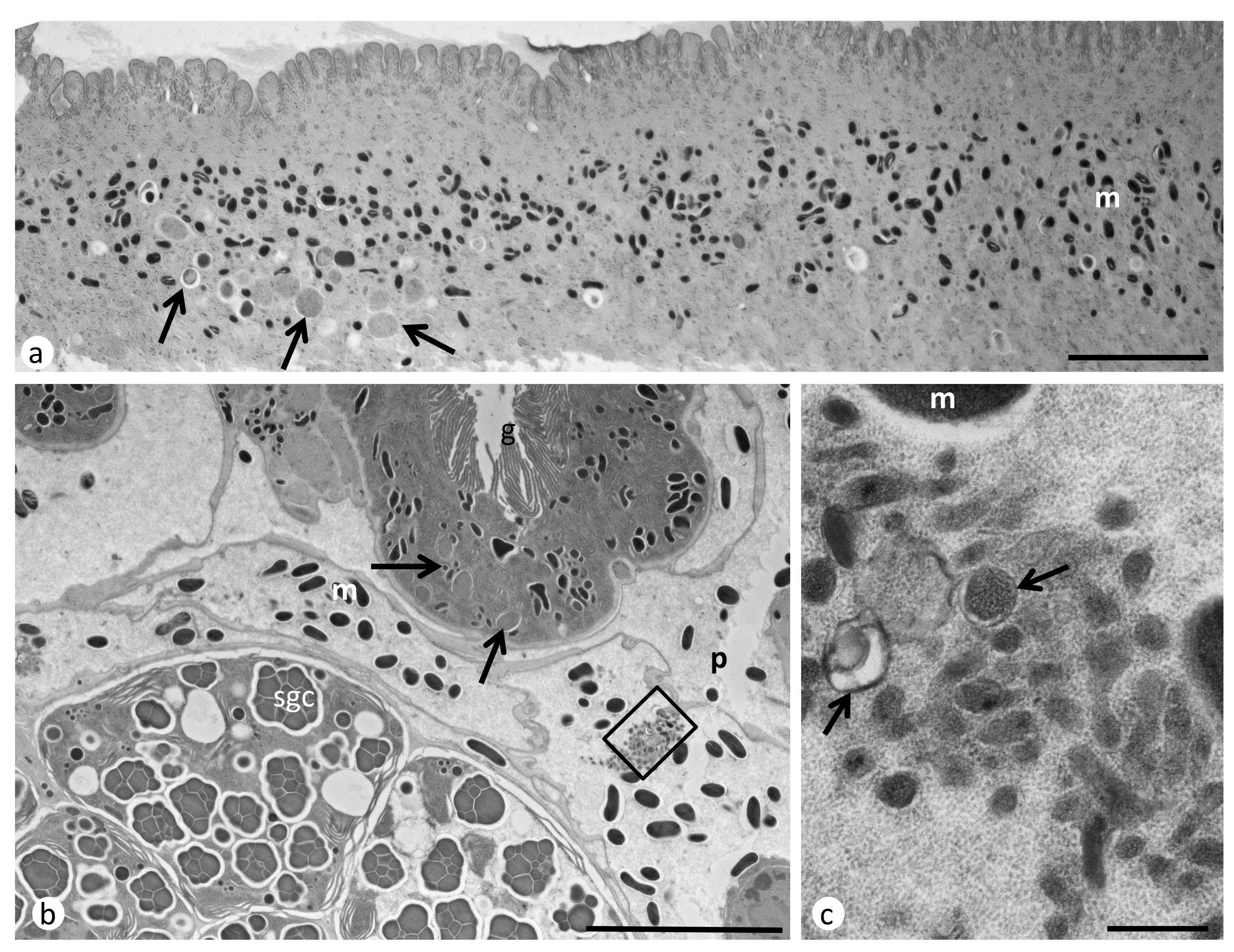

Supplement: Supplementary file 4 — TEM of HPF/FS fixed P. elegans. a Overview of a cross-section of the tegument showing a lose cluster of endobacteria (arrows) in one area, but no endobacteria in other areas. b Large endobacteria (arrows) are localized in the wall of the gut, while endobacteria are mostly absent in adjacent tissues. Note the cluster of small structures (boxed area) in the parenchyma. c Magnification of boxed area from b shows a few small endobacteria (arrows) with typical membrane structures among electron dense structures without pronounced membranes. Abbreviations: m, mitochondrion; sgc, shell globule cluster. Scale-bars: a, b, 5 μm; c 200 nm. (TIF 6120 kb) [file 13071_2017_2123_MOESM4_ESM.tif]

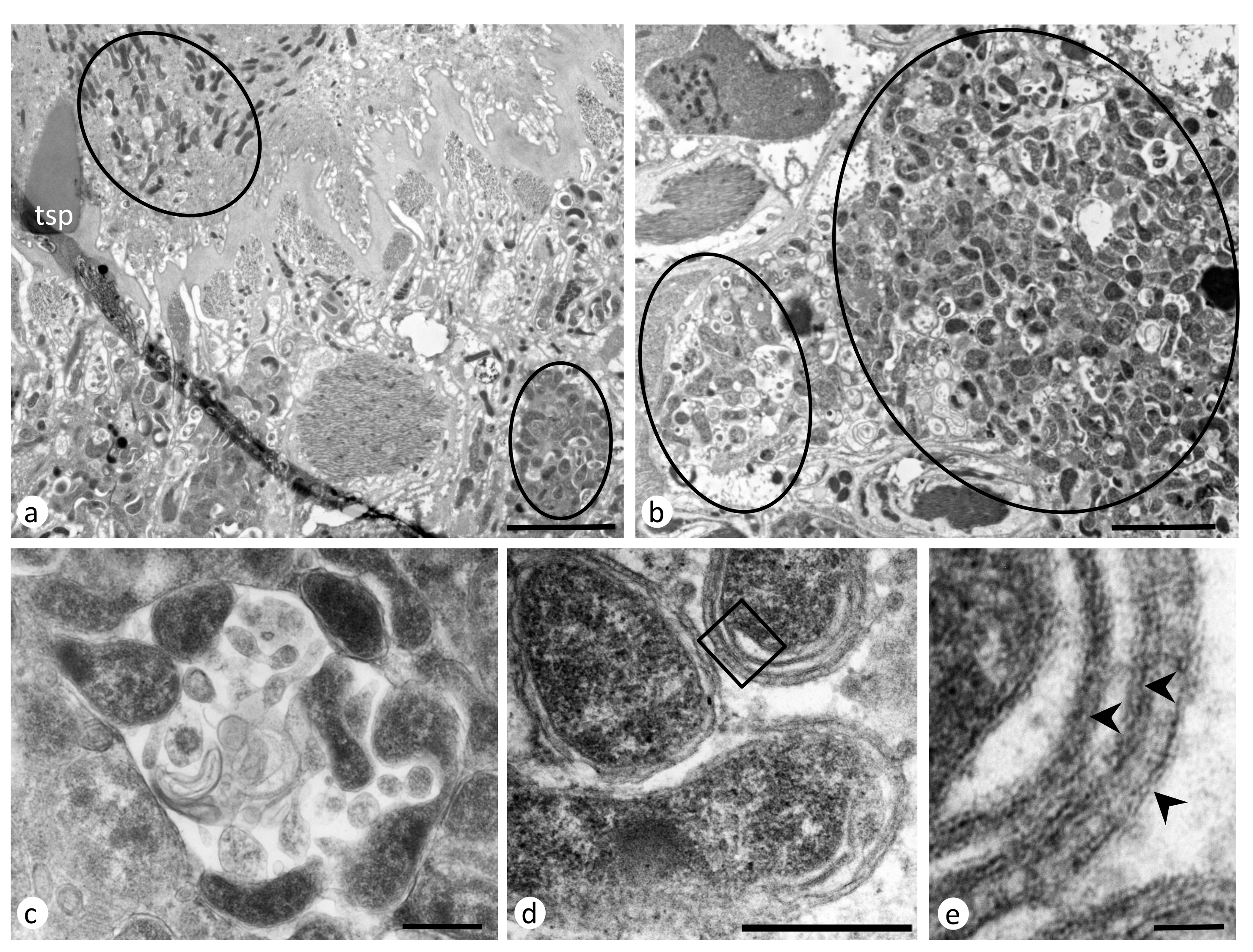

Supplement: Supplementary file 5 — TEM of chemically fixed P. elegans. a Large clusters of endobacteria in the tegument (circles) and the cytons. b Large clusters of endobacteria (circles) in the subtegument. c Magnification of b showing pleomorphic endobacteria without well preserved membranes. d Three endobacteria with multiple and well preserved membranes. e Magnification of boxed area of d showing the well preserved membrane structure. Abbreviation: tsp, tegumental spine. Scale-bars: a, b, 2 μm; c-e, 500 nm. (TIF 6604 kb) [file 13071_2017_2123_MOESM5_ESM.tif]

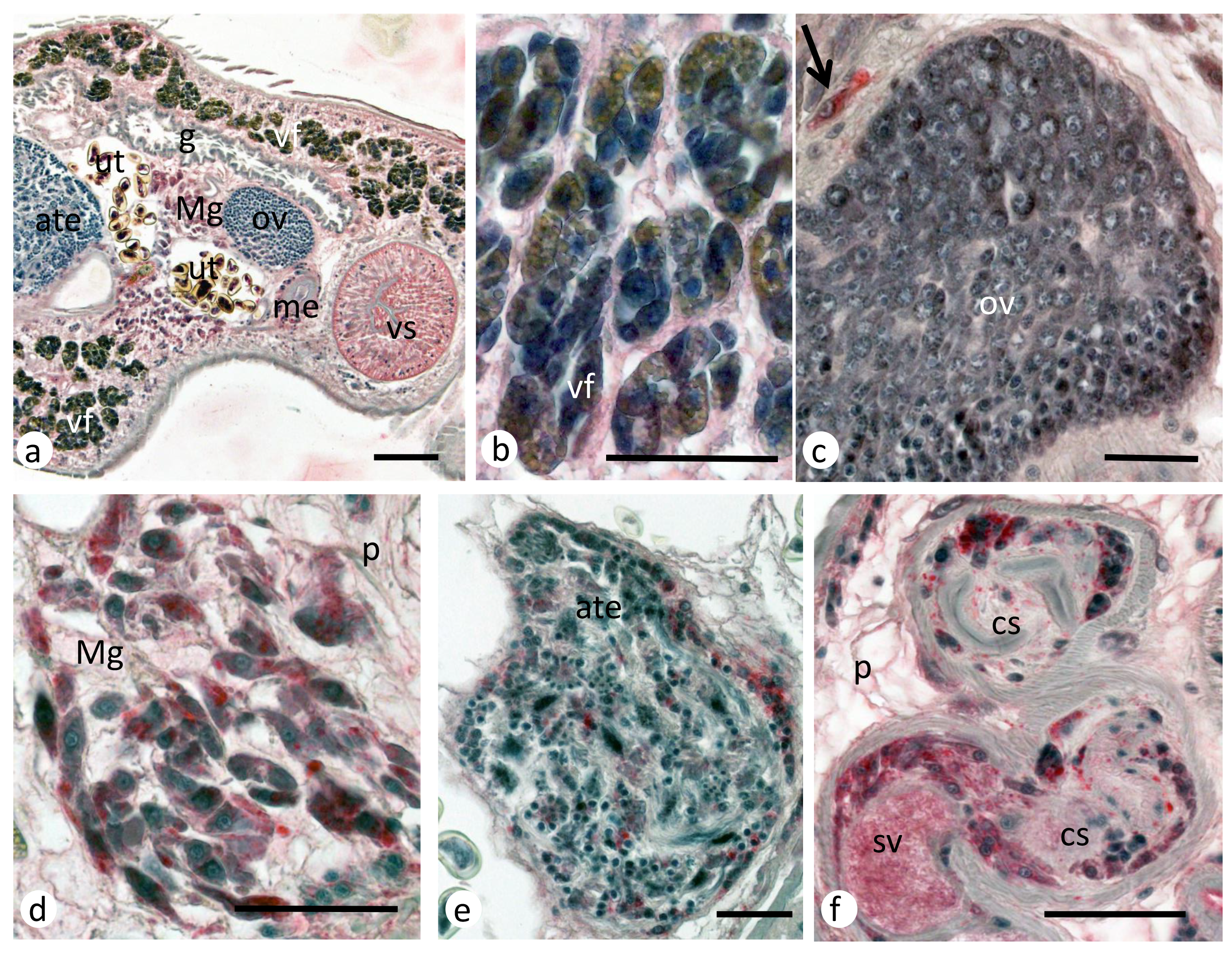

Supplement: Supplementary file 6 — APAAP staining for endobacteria in P. elegans using polyclonal antisera against rPeNsp-3. a Longitudinal section near the ventral sucker showing diffuse red staining for endobacteria in many tissue types. b Light pink background around the vitelline follicles (compare to Fig. 8a). c Similar to b ovary appears to be endobacteria free (no red stain), notice the red staining outside of the ovary (arrow). d Intense red staining for endobacteria in the Mehlis’ gland where density of Neorickettsia is high (compare to Fig. 8a). e Intense, but limited red staining for endobacteria in the testis (compare to Fig. 9a). f Intense red staining for endobacteria in the seminal vesicle and cirrus sac (compare to Fig. 9b). Abbreviations: ate, anterior testis; cs, cirrus-sac; g, gut; me, metraterm; Mg, Mehlis’ gland; ov, ovary; p, parenchym; sv, seminal vesicle; ut, uterus; vf, vitelline follicle; vs, ventral sucker. Scale-bars: a, c, e, f 100 μm; b, d, 25 μm. (TIF 7473 kb) [file 13071_2017_2123_MOESM6_ESM.tif]

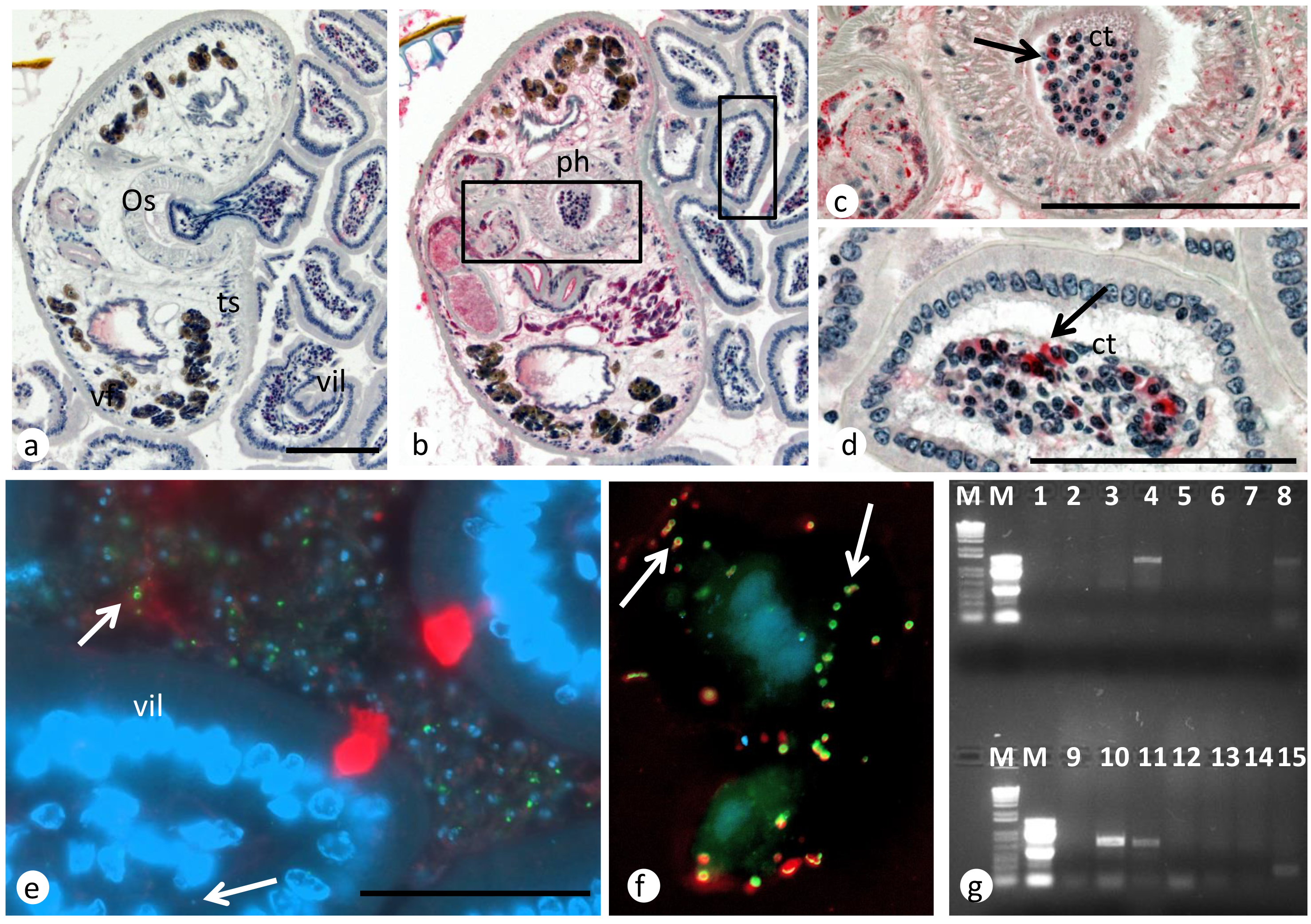

Supplement: Supplementary file 7 — Detection of Neorickettsia outside P. elegans (a-d APAAP staining, e-f immunofluorescence). a Adult P. elegans in the intestine of a hamster, pre-immune serum. b Consecutive section to a but stained with anti-sera against PeNsp-3. c Magnification of b showing connective tissue of the gut within the pharynx of P. elegans. Note single red cells (arrow) positive for PeNsp-3. d Another magnification of b showing single red cells (arrow) positive for PeNsp-3 in connective tissue within the hamster villi. e Green labeling for PeNsp-3 of bacteria (arrow) within the intestinal mucus or connective tissue within the villi. f High resolution, donut-shaped labeling for PeNsp-3 of bacteria (arrow) in the mucus. g Conventional PCR detection of Neorickettsia targeting 16S rDNA. Lane M: Marker; Lane 9: no template; Lanes 10, 11: Neorickettsia positive trematodes in field-collected snails; Lanes 1–4: P. elegans-infected hamster 1; Lanes 5–8: P. elegans-infected hamster 2; Lanes 12–15: uninfected hamster. Lanes 1, 5, 12: heart tissue; Lanes 2, 6, 13: kidney tissue; Lanes 3, 7, 14: spleen tissue; Lanes 4, 8, 15: small intestine tissue. Abbreviations: ct, connective tissue; os, oral sucker; ph, pharynx; ts, tegumental syncytium; vil, villi of the intestine. Scale-bars: 100 μm. (TIF 7014 kb) [file 13071_2017_2123_MOESM7_ESM.tif]
