# Supplementary material for: Ultrastructure and localization of Neorickettsia in adult digenean trematodes provides novel insights into helminth-endobacteria interaction
Source: Parasit Vectors. 2017 Apr 13;10:177. doi: 10.1186/s13071-017-2123-7 (PMC5390476; doi:10.1186/s13071-017-2123-7)
Supplement: Supplementary file 2 — Primers and probes used to detect Neorickettsia DNA and P.elegans DNA in various tissue samples by qPCR. (DOCX 15 kb) [file 13071_2017_2123_MOESM2_ESM.docx]

Table S1. Primers and probes used to detect *Neorickettsia* DNA and *P.elegans* DNA in various tissue samples by qPCR.

| Target |  |  | Remarks |
| --- | --- | --- | --- |
| *P. ele*gans 28S rDNA |  |  | GenBank KF556678.1 |
|  | Forward | 5’-TCCGAGTTTGGTTGTCATGTT-3’ | Position 806 to 826 |
|  | Reverse | 5’-ACTGTTGGCCCTTGGTTTAG-3’ | Position 889 to 908 |
|  | Probe | 5’-/56FAM/ CACGCAACC/ZEN/ACCCACAGAACAAAC/3IABkFQ/-3’ | Position 881 to 858 |
| *Neorickettsia* surface protein-3 |  |  | GenBank  KX082665.1 |
|  | Forward | 5’-GGTGCACTCGGGTACTATTT-3’ | Position 238 to 257 |
|  | Reverse | 5’-CTGAAGCAACAGGAGTTGTAGA-3’ | Position 325 to 346 |
|  | Probe | 5’-/56FAM/ AGTGTACAC/ZEN/AAGGTCGCTGCTGAA/3IABkFQ/-3’ | Position 286 to  309 |
