# Supplementary material for: Ultrastructure and localization of Neorickettsia in adult digenean trematodes provides novel insights into helminth-endobacteria interaction
Source: Parasit Vectors. 2017 Apr 13;10:177. doi: 10.1186/s13071-017-2123-7 (PMC5390476; doi:10.1186/s13071-017-2123-7)
Supplement: Supplementary file 8 — Comparison of qPCR amplification (mean cycle threshold values, CT) of P.elegans DNA and Neorickettsia DNA from 20 mg of various tissues. Both targets were only detected in isolated P. elegans worms and in the small intestine from hamsters infected with P. elegans (but without adult trematodes present). Significantly more Neorickettsia DNA relative to trematode DNA was found in the intestine. (DOCX 14 kb) [file 13071_2017_2123_MOESM8_ESM.docx]

Table S2. Comparison of qPCR amplification (mean cycle threshold values, CT) of *P.elegans* DNA and *Neorickettsia* DNA from 20 mg of various tissues. Both targets were only detected in isolated *P. elegans* worms and in the small intestine from hamsters infected with *P. elegans* (but without adult trematodes present).

| Specimen | *P.elegans* qPCR (ITS) | | *Neorickettsia* qPCR (PeNsp-3) | |
| --- | --- | --- | --- | --- |
|  | Mean CT | 95% CI | Mean CT | 95% CI |
| Isloated *P. elegans* worms | 14.95 | 14.79-15.11 | 19.38 | 19.19-19.57 |
| Gut of hamsters infected with *P. elegans** | 28.18 | 23.84-32.53 | 28.93 | 28.08-29.79 |
| Spleen of hamsters infected with *P. elegans** | >40 | - | >40 | - |
| Kidney of hamsters infected with *P. elegans** | >40 | - | >40 | - |
| Heart of hamsters infected with *P. elegans** | >40 | - | >40 | - |

*But without trematodes
